# A gap in the prognostic score for atherosclerotic renovascular disease: the role of statins

**DOI:** 10.1590/2175-8239-JBN-2025-E018en

**Published:** 2025-10-10

**Authors:** Rodrigo Peixoto Campos

**Affiliations:** 1 Universidade Federal do Paraná, Curitiba, PR, Brazil.; 2 Pontifícia Universidade Católica do Paraná, Curitiba, PR, Brazil.

Atherosclerotic renovascular disease (ARVD) may present asymptomatically, as an incidental finding of renal artery stenosis (>50% luminal reduction). However, its more severe clinical manifestations – such as renovascular hypertension, ischemic nephropathy, and decompensation of cardiovascular diseases (including heart failure and stroke) – require greater attention. It is a highly prevalent vascular condition, particularly among the elderly, affecting approximately 6.8% of the population over 65 years of age^1^.

ARVD is often associated with other comorbidities—such as systemic arterial hypertension, diabetes mellitus, chronic kidney disease, peripheral arterial disease, coronary artery disease, and heart failure—and carries a poor prognosis. Among elderly patients with ARVD, according to Medicare data (USA), the annual rates of stroke, acute coronary syndrome, heart failure, and mortality were 18%, 30%, 19%, and 17%, respectively^2^. In the CORAL study (Stenting and Medical Therapy for Atherosclerotic Renal-Artery Stenosis), after a mean follow-up of 43 months, 35.4% of patients reached the primary outcome. Among the secondary outcomes, the following were observed: acute myocardial infarction in 8.3%, stroke in 4.2%, progression of chronic kidney disease in 17.8%, hospitalization for heart failure in 8.4%, and overall mortality in 14.9%^3^. ARVD should therefore be understood within the context of systemic atherosclerotic disease.

In order to assess overall and cardiovascular event-free survival at 1, 5, and 10 years, Vassalo et al. developed a prognostic score based on a cohort of 872 ARVD patients, followed between 1986 and 2014. The model included the following variables: age, estimated glomerular filtration rate (eGFR), history of renal revascularization, acute myocardial infarction, presence of heart failure, and peripheral arterial disease^4^. As most patients were included in a pre-statin era (considering that lovastatin was introduced in the US in 1987), the impact of these medications was not considered in the modeling.

In the study published in this issue of the Brazilian Journal of Nephrology, Salome et al. contribute by applying the Vassalo score to a contemporary cohort of 103 patients with ARVD followed for more than 12 years^5^. The main finding was a marked discrepancy between observed and predicted survival in the subgroup of patients taking statins. These individuals showed significantly higher survival rates than estimated by the model, with differences of 9% in the first year (0.95 vs. 0.87), 95% at 5 years (0.88 vs. 0.45), and 380% at 10 years (0.72 vs. 0.15). Conversely, among those not taking statins, the observed and predicted survival curves were quite similar.

The statin group had a higher prevalence of dyslipidemia, coronary artery disease, and use of angiotensin-converting enzyme (ACE) inhibitors or angiotensin receptor blockers (ARBs). In addition, the mean baseline eGFR was higher in this group (42 vs. 33.6 mL/min/1.73m^2^). Although this difference did not reach statistical significance, it is plausible that it contributed to the improved outcomes, considering that both reduced eGFR and its decline over time are associated with increased cardiovascular risk and mortality^6,7^. Thus, baseline eGFR may have acted as an important confounding factor in the results. Another relevant aspect to be considered, as rightly pointed out by the authors, was the time difference between the original cohort used in the Vassalo score (1980s) and the more recent cohort initiated in the 1990s – when statins, ACE inhibitors, and beta-blockers were already widely used.

In recent years, international guidelines, such as those from Kidney Disease: Improving Global Outcomes (KDIGO) and the European Renal Association, have recommended the use of statins in ARVD, with the aim of reducing both the progression of atherosclerotic stenosis and cardiovascular risk^8,9^. In a Canadian cohort of 4,040 elderly patients with ARVD, statin use was associated with a 49% reduction in the risk of cardiorenal events (HR 0.51; 95% CI: 0.46–0.57).

In addition to statins, treatment of ARVD should include strict blood pressure control (preferably with ACE inhibitors/ARBs), glycemic control, smoking cessation, and the use of low-dose aspirin. Revascularization by percutaneous transluminal angioplasty, with or without stenting, remains controversial. However, patients with resistant hypertension, heart failure, episodes of acute pulmonary edema, or rapid deterioration of kidney function may benefit from this procedure. Stenoses >70% and ultrasound findings such as kidney >8 cm, cortex >0.5 cm, and resistance index >0.8 are also predictors of good response to revascularization.

In summary, statins are drugs with robust evidence in the prevention of cardiovascular events, and their role in ARVD should be widely acknowledged. The study by Salome et al.^5^ demonstrated that prognostic models that do not account for the use of these medications may underestimate patient survival. Thus, the elements discussed reinforce the importance of revisiting and updating prognostic scores in light of the clinical and therapeutic changes that occur over time. The incorporation of these factors, as well as the findings reported by Salome et al^5^, in the prognostic assessment of patients with ARVD further underscores this need.

## Data Availability

No new data were generated or analyzed in this study.
