# Supplementary material for: A gap in the prognostic score for atherosclerotic renovascular disease: the role of statins
Source: J Bras Nefrol. 2025 Oct 10;47(4):e2025E018. doi: 10.1590/2175-8239-JBN-2025-E018en (PMC12513559; doi:10.1590/2175-8239-JBN-2025-E018en)
Supplement: Translation

# Translation: Uma lacuna no escore prognóstico da doença renovascular aterosclerótica: o papel das estatinas

**DOI:** 10.1590/2175-8239-JBN-2025-E018pt

**Authors:** Rodrigo Peixoto Campos

**Affiliations:** 1 Universidade Federal do Paraná, Curitiba, PR, Brasil.; 2 Pontifícia Universidade Católica do Paraná, Curitiba, PR, Brasil.

A doença renovascular aterosclerótica (DRVA) pode se apresentar de forma assintomática, como um achado incidental de estenose da artéria renal (>50% de redução luminal). No entanto, suas manifestações clínicas mais graves — como hipertensão renovascular, nefropatia isquêmica e descompensação de doenças cardiovasculares (incluindo insuficiência cardíaca e acidente vascular cerebral) — demandam maior atenção. Trata-se de uma condição vascular altamente prevalente, especialmente entre idosos, acometendo cerca de 6,8% da população acima de 65 anos^1^.

A DRVA é frequentemente associada a outras comorbidades — como hipertensão arterial sistêmica, diabetes mellitus, doença renal crônica, doença arterial periférica, doença arterial coronariana e insuficiência cardíaca — e apresenta prognóstico desfavorável. Entre idosos com DRVA, de acordo com dados do Medicare (EUA), as taxas anuais de acidente vascular cerebral, síndrome coronariana aguda, insuficiência cardíaca e mortalidade foram de 18%, 30%, 19% e 17%, respectivamente^2^. No estudo CORAL (*Stenting and Medical Therapy for Atherosclerotic Renal-Artery Stenosis*), após um seguimento médio de 43 meses, 35,4% dos pacientes atingiram o desfecho primário. Entre os desfechos secundários, observaram-se: infarto agudo do miocárdio em 8,3%, acidente vascular cerebral em 4,2%, progressão da doença renal crônica em 17,8%, internação por insuficiência cardíaca em 8,4% e mortalidade geral em 14,9%^3^. A DRVA, portanto, deve ser compreendida no contexto da doença aterosclerótica sistêmica.

Com o objetivo de avaliar a sobrevida global e livre de eventos cardiovasculares em 1, 5 e 10 anos, Vassalo et al. desenvolveram um escore prognóstico a partir de uma coorte de 872 pacientes com DRVA, acompanhados entre 1986 e 2014. O modelo contemplou as seguintes variáveis: idade, taxa de filtração glomerular estimada (TFGe), histórico de revascularização renal, infarto agudo do miocárdio, presença de insuficiência cardíaca e doença arterial obstrutiva periférica^4^. Como grande parte dos pacientes foi incluída em uma era pré-estatinas (considerando que a lovastatina foi lançada nos EUA em 1987), o impacto dessas medicações não foi considerado na modelagem.

No estudo publicado nesta edição do *Brazilian Journal of Nephrology*, Salome et al. contribuem ao aplicar o escore de Vassalo em uma coorte contemporânea de 103 pacientes com DRVA acompanhados por mais de 12 anos^5^. A principal constatação foi uma discrepância marcante entre a sobrevida observada e predita no subgrupo de pacientes em uso de estatina. Esses indivíduos apresentaram sobrevida significativamente superior à estimada pelo modelo, com diferenças de 9% no primeiro ano (0,95 vs. 0,87), 95% em 5 anos (0,88 vs. 0,45) e 380% em 10 anos (0,72 vs. 0,15). Por outro lado, entre os que não utilizavam estatinas, as curvas de sobrevida observada e esperada foram bastante semelhantes.

O grupo em uso de estatinas apresentou maior prevalência de dislipidemia, doença arterial coronariana e uso de inibidores da enzima conversora de angiotensina (IECA) ou bloqueadores do receptor de angiotensina (BRA). Além disso, a TFGe inicial média era superior nesse grupo (42 vs. 33,6 mL/min/1,73m^2^). Embora essa diferença não tenha atingido significância estatística, é plausível que tenha contribuído para os melhores desfechos, considerando que tanto a TFGe reduzida quanto seu declínio ao longo do tempo estão associados a maior risco cardiovascular e à mortalidade^6,7^. Assim, a TFGe inicial pode ter atuado como importante fator de confusão nos resultados. Outro aspecto relevante a ser considerado foi a diferença temporal, como bem apontado pelos autores, entre a coorte original utilizada no escore de Vassalo (década de 1980) e a coorte mais contemporânea, iniciada na década de 1990, em que estatinas, IECAs e betabloqueadores já eram amplamente utilizados.

Nos últimos anos, diretrizes internacionais, como as do *Kidney Disease: Improving Global Outcomes* (KDIGO) e da *European Renal Association*, passaram a recomendar o uso de estatinas na DRVA, com o objetivo de reduzir tanto a progressão da estenose aterosclerótica quanto o risco cardiovascular^8,9^. Em uma coorte canadense com 4.040 idosos com DRVA, o uso de estatinas foi associado a uma redução de 49% no risco de eventos cardiorrenais (HR 0,51; IC 95%: 0,46–0,57).

Além das estatinas, o tratamento da DRVA deve incluir o controle rigoroso da pressão arterial (preferencialmente com IECA/BRA), o controle glicêmico, a cessação do tabagismo e o uso de aspirina em baixa dose. A revascularização por angioplastia transluminal percutânea, com ou sem *stent*, permanece controversa. No entanto, pacientes com hipertensão resistente, insuficiência cardíaca, episódios de edema agudo de pulmão ou deterioração rápida da função renal podem se beneficiar desse procedimento. Estenoses >70% e achados ultrassonográficos, como rim >8 cm, córtex >0,5 cm e índice de resistência >0,8 também são preditores de boa resposta à revascularização.

Em síntese, as estatinas são fármacos com sólida evidência na prevenção de eventos cardiovasculares, e seu papel na DRVA deve ser amplamente reconhecido. O estudo de Salome et al.^5^ demonstrou que modelos prognósticos que não consideram o uso dessas medicações podem subestimar a sobrevida dos pacientes. Assim, os elementos discutidos reforçam a importância de revisitar e atualizar escores prognósticos à luz das transformações clínicas e terapêuticas que ocorrem ao longo do tempo. A incorporação desses fatores, bem como os achados reportados por Salome et al.^5^, na avaliação prognóstica de pacientes com DRVA, reforçam essa necessidade.

## Data Availability

Nenhum dado novo foi gerado ou analisado neste estudo.
